# Association of the dietary-behavioral risk index with metabolic syndrome and its components: a cross-sectional study in rural China

**DOI:** 10.3389/fendo.2026.1895259

**Published:** 2026-09-04

**Authors:** Jiangwei Qiu, Jiao Cui, Kexin Chen, Jiaxing Zhang, Yuhong Zhang, Yi Zhao

**Affiliations:** 1 School of Public Health, Ningxia Medical University, Yinchuan, China; 2 Key Laboratory of Environmental Factors and Chronic Disease Control, Ningxia Medical University, Yinchuan, China; 3 School of Nursing, Ningxia Medical University, Yinchuan, China

**Keywords:** diet quality, dietary-behavioral risk index, lifestyle, metabolic syndrome, rural population

## Abstract

**Background:**

Metabolic syndrome (MetS) has become a growing public health concern. However, systematic assessment of combined lifestyle and multidimensional dietary factors in rural Chinese populations remains limited. This study aimed to investigate the associations of an obesity-centered lifestyle score (OLS), Diet Quality Index-International (DQI-I), and a newly constructed dietary-behavioral risk index (DBRI) with MetS and its components in a rural Chinese population.

**Methods:**

This cross-sectional study included 14,269 residents from the Northwest China Natural Population Cohort (CNC-NX). DBRI was constructed using the Alkire-Foster dual cut-off approach and categorized into quartiles. Spearman correlation and multivariate logistic regression were used to examine the associations of OLS, DQI-I, and DBRI with MetS.

**Results:**

Both OLS and DBRI were positively correlated with MetS prevalence (both P<0.001). DBRI showed a significant dose-response association with MetS, with the highest quartile having an odds ratio of 2.61 (95% CI: 2.29-2.98) compared with the lowest. A nonlinear relationship was observed using restricted cubic splines (P for nonlinear=0.013), with a threshold effect near DBRI≈0.5. The total DQI-I score was not significantly associated with MetS.

**Conclusion:**

DBRI, a composite index integrating lifestyle and dietary quality, was significantly associated with MetS in a dose-response manner, suggesting its potential utility in risk stratification. Public health interventions should focus on dietary optimization and long-term promotion of obesity-related lifestyle habits.

## Introduction

Metabolic syndrome (MetS) is a complex metabolic disorder characterized by abdominal obesity, hypertension, hyperglycemia, and dyslipidemia. It is a major risk factor for cardiovascular and cerebrovascular diseases as well as type 2 diabetes mellitus (1–3). The prevalence of MetS has been increasing globally, imposing a substantial burden on public health (4, 5). In recent years, rapid socioeconomic transformation in China has led to marked changes in lifestyle and dietary patterns, and the prevalence of MetS has risen accordingly, becoming a prominent health issue (6, 7).

Although numerous studies have examined the associations of single lifestyle factors (e.g., diet, physical activity) or obesity indicators with MetS, they have limited ability to reflect the combined effects of multiple behavioral factors. A recent meta-analysis showed that individuals with the highest healthy lifestyle score had a 43% lower risk of MetS compared with those with the lowest score, highlighting the value of comprehensive lifestyle assessment (8). Another study in an urban population further found a combined effect of unhealthy lifestyle and unhealthy dietary habits on MetS, with lifestyle possibly masking the association between dietary habits and MetS (9). However, most of this evidence comes from urban or general populations. In rural China, lifestyle behaviors (e.g., physical activity patterns, smoking, and alcohol consumption) often intertwine with local dietary culture and jointly influence health outcomes. Currently, studies that combine lifestyle scores with diet quality assessment in rural Chinese populations remain insufficient. Existing research has either failed to capture the holistic nature of lifestyle or lacked large-scale in-depth analyses tailored to the specific living environment of rural areas.

Rural China is undergoing a profound transition. Regarding lifestyle, urbanization has led to decreased physical activity levels and increased sedentary time among middle-aged and older adults; at the same time, the return of aging migrant workers and demographic shifts are reshaping the lifestyle of rural residents (10, 11). Regarding dietary patterns, the dietary structure is shifting from a traditional plant-based diet to a Western-style diet rich in animal products. Consumption of meat, eggs, and milk has increased, while intake of traditional healthy foods such as coarse grains and legumes has decreased, resulting in insufficient dietary diversity (12, 13). Against this background, the prevention and control of MetS in rural areas faces multiple challenges: low health literacy, limited intervention strategies, and scarce primary healthcare resources (14, 15). Thus, given that multi-modal lifestyle interventions are superior to single-component interventions in reversing metabolic syndrome (16), there is an urgent need to develop comprehensive risk assessment tools suitable for rural populations to identify high-risk individuals and guide stratified intervention strategies.

The dual transition in lifestyle and dietary patterns suggests that single behavioral factors cannot fully explain the risk of MetS in rural populations (8, 9). Although some studies have explored comprehensive lifestyle scores - for example, Bai et al. found that a healthy lifestyle significantly reduced the risk of comorbidity in a rural older diabetic population (17) - most of these studies did not simultaneously integrate diet quality assessment. Research that combines lifestyle scores with diet quality assessment in rural Chinese populations remains relatively limited (18). Obesity is a key risk factor for metabolic diseases (19). In this study, we constructed an obesity-centered lifestyle score (OLS), used the Diet Quality Index-International (DQI-I) to assess dietary nutrition, and combined the two using the Alkire-Foster dual cut-off approach to create the Dietary-Behavioral Risk Index (DBRI), which comprehensively reflects the overall status of lifestyle and diet quality. Through a cross-sectional survey, we aim to investigate the associations of these indices with MetS and its components, thereby providing a scientific basis for comprehensive intervention strategies for MetS in rural Ningxia.

## Methods

### Study design and population

This study was a cross-sectional study based on baseline data from the second phase of the Northwest China Natural Population Cohort - Ningxia Project (CNC-NX). Two towns in Qingtongxia City and Pingluo County, Ningxia, were selected as survey sites. The baseline survey was conducted from March 2018 to May 2019. Stratified cluster sampling was used to collect biological samples from participants, and physical examinations and questionnaire surveys were completed. After a rigorous inclusion and exclusion process, 15,802 residents aged 35–74 years were finally included in the baseline cohort database. This study was approved by the Ethics Committee of Ningxia Medical University (approval number: 2018-012), and all participants signed informed consent forms after fully understanding the purpose of the study.

**Figure 1:**
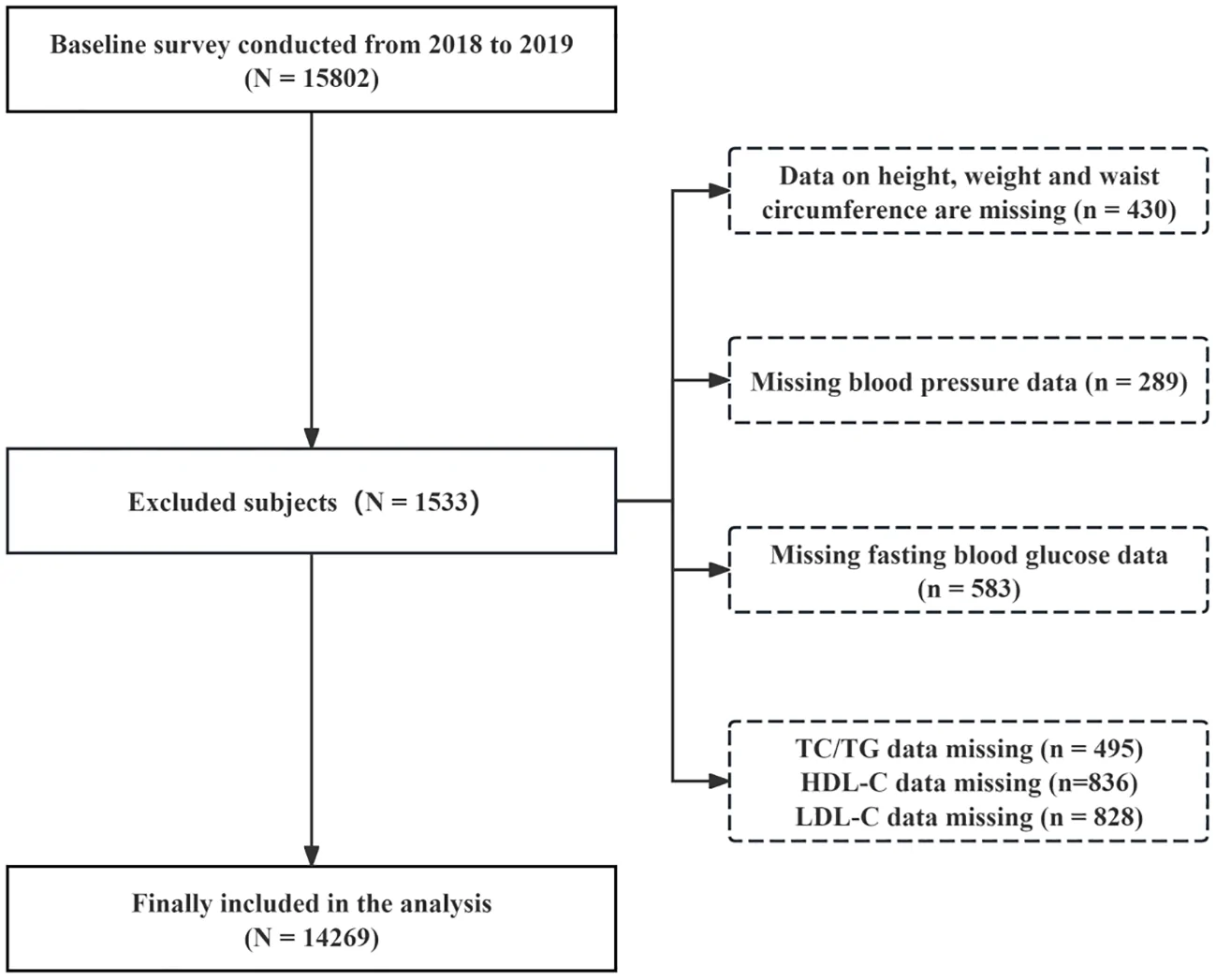
Flowchart of participant selection (CNC-NX, 2018-2019). After applying the baseline survey inclusion and exclusion criteria, a total of 15,802 participants were enrolled; after excluding 1,533 with missing data on physical examinations and biochemical tests, 14,269 were included in the final analysis. The numbers of missing data for each variable are shown in the corresponding boxes.

The inclusion and exclusion criteria for the baseline survey were as follows. Inclusion criteria: (i) age 35–74 years and having lived locally for at least three generations; (ii) signed informed consent. Exclusion criteria: (i) patients with severe infectious diseases, mental disorders, tumors, or other major diseases during the survey period; (ii) patients with diseases that significantly affect normal dietary intake; (iii) participants who did not sign the informed consent. We excluded 1,533 participants with missing physical examination and biochemical test data and finally included 14,269 participants for analysis, as shown in **Figure 1**.

### Data collection and measurements

Trained investigators conducted face-to-face questionnaire surveys. The questionnaire covered the following aspects of the participants: demographic characteristics (sex, ethnicity, marital status, education level), lifestyle behaviors (smoking habits, alcohol consumption, frequency of physical activity), medical history (hypertension, diabetes, coronary heart disease), medication use (antihypertensive and glucose-lowering drugs), and dietary nutrition survey (20).

Physical examinations included height, weight, waist circumference, hip circumference, blood pressure, and other indices. Body mass index (BMI) and blood pressure were calculated. Height was measured using a stadiometer. Weight, waist circumference, and hip circumference were measured using a bioelectrical impedance analyzer (Inbody-370s, Seoul, Republic of Korea). Participants were asked to remove coats, shoes, socks, and metal accessories, stand barefoot on the scale, hold the handle, and remain still until the measurement was completed. Systolic blood pressure (SBP) and diastolic blood pressure (DBP) were measured using an electronic sphygmomanometer (OMRON-7124).

The study was conducted in township health centers. Fasting peripheral venous blood samples were collected in the morning. Participants were required to fast for at least 8 hours and to avoid strenuous exercise before blood collection. Professional nurses followed standardized procedures strictly. Immediately after collection, the time and participant ID were recorded, followed by centrifugation and sample aliquoting. Centrifugation was performed on site. The samples were refrigerated and then transported to the health center laboratory. After being frozen, the samples were transferred to a biobank and stored in a -80 °C freezer. Fasting plasma glucose (FPG), total cholesterol (TC), triglycerides (TG), high-density lipoprotein cholesterol (HDL-C), and low-density lipoprotein cholesterol (LDL-C) were measured using an automated biochemical analyzer (CS-T3000, provided by the local health center).

### Disease definitions

Hypertension was diagnosed according to the Chinese Guidelines for the Prevention and Treatment of Hypertension (2024 revision) (21): either office blood pressure ≥140/90 mmHg on three separate occasions without antihypertensive medication, home blood pressure ≥135/85 mmHg over 5–7 consecutive days, or 24-h ambulatory blood pressure ≥130/80 mmHg; participants with a previous history of hypertension were also considered as having hypertension. Diabetes mellitus was diagnosed according to the Chinese Guidelines for the Prevention and Treatment of Diabetes (2024) (22): typical diabetic symptoms plus random blood glucose ≥11.1 mmol/L, or fasting blood glucose ≥7.0 mmol/L, or 2-h postload glucose ≥11.1 mmol/L during an oral glucose tolerance test (OGTT), or glycated hemoglobin (HbA1c) ≥6.5%. In the absence of typical symptoms, confirmation required a repeat test on another day.

Metabolic syndrome (MetS) was defined as the presence of at least three of the following criteria (22): (i) abdominal obesity (central obesity): waist circumference ≥90 cm in men or ≥85 cm in women; (ii) hyperglycemia: fasting plasma glucose ≥6.1 mmol/L or 2-h OGTT glucose ≥7.8 mmol/L, and/or a prior diagnosis of diabetes with ongoing treatment; (iii) elevated blood pressure: blood pressure ≥130/85 mmHg (1 mmHg = 0.133 kPa) and/or a previous diagnosis of hypertension with treatment; (iv) fasting triglycerides (TG) ≥1.70 mmol/L; (v) fasting high-density lipoprotein cholesterol (HDL-C) <1.04 mmol/L. In addition, we calculated a metabolic health score as previously described (23). For each of the five MetS components, a positive result was assigned 1 point and a negative result 0 points; the total score ranged from 0 to 5, with higher scores indicating worse health status. Body mass index (BMI) was calculated as weight (kg) divided by height squared (m²).

### Index construction

#### Obesity-centered lifestyle score

The obesity-centered lifestyle score (OLS) was calculated based on the method of Yun et al., using questionnaire data that covered behavioral lifestyle factors including smoking status, alcohol consumption habits, and physical activity level (24). This score adopted a binomial approach, assigning 1 point for each of the following positive statuses: overweight or obesity (BMI > 25 kg/m²), smoking (including current smokers and those who had quit for less than 10 years), alcohol consumption (two drinks per day for men or one drink per day for women), and physical inactivity (exercising less than three times per week). If all items were negative, no point was assigned. Thus, the OLS ranged from 0 to 4 points.

#### Diet quality index-international

The Diet Quality Index-International (DQI-I) was used to assess the quality of dietary intake. Dietary data were obtained from the baseline survey of the second phase of the CNC-NX cohort (2018-2019). Dietary intake was assessed using a validated semi-quantitative food frequency questionnaire (SFFQ), which had demonstrated good reliability and validity in a population at high risk for cardiovascular and cerebrovascular diseases in Ningxia (25). Given the homogeneity in dietary culture between individuals with metabolic syndrome and that population, the SFFQ was considered suitable for this study. The SFFQ collected information on dietary intake over the previous year, covering 11 major categories (e.g., staple foods, animal-based foods, plant-based foods, other foods, soft drinks, local specialty foods, and oils and fats) comprising 67 food items.

As a comprehensive assessment tool, the DQI-I can more accurately reflect dietary nutrition status than traditional nutrient-based assessment methods. This index was developed by Kim et al. in 2003 (26) and uses a 100-point scale to score diet quality, with higher scores indicating better diet quality. The assessment includes four components: dietary diversity (0–20 points), adequacy (0–40 points), moderation (0–30 points), and overall balance (0–10 points). The DQI-I is not only useful for cross-national comparisons of diet quality and for monitoring dietary health status across countries, but also provides useful information for nutritional interventions and education programs (27) (**Supplementary Table 6**).

#### Dietary-behavioral risk index

The Dietary-Behavioral Risk Index (DBRI) was constructed using the Alkire-Foster dual cut-off approach (28). This method identifies patterns of risk aggregation across multiple indicators and determines the overall risk status of an individual based on two thresholds: the within-dimension deprivation cutoff and the cross-dimensional risk cutoff (k). This approach was chosen because dietary-behavioral risk is characterized by the clustering of multiple factors. The Alkire-Foster method simultaneously accounts for the cumulative effects of multiple risk factors and allows for partial compensation across dimensions, making it more suitable for capturing real-world exposure patterns than single-index weighting(**Supplementary Table 9**).

The DBRI system comprises two dimensions: lifestyle risk (alcohol consumption, smoking, overweight or obesity, and physical inactivity) and dietary quality risk (dietary diversity, dietary adequacy, dietary moderation, and overall balance). For each lifestyle indicator, the deprivation cutoff was defined as the presence of the risk factor (coded as 1). For each dietary quality indicator, the deprivation cutoff was set at the 40th percentile of the DQI-I score, with values below P_40_ coded as 1 (29). This cutoff follows the common practice in previous studies of defining “lower diet quality” as the tail of the distribution (28, 29). At the same time, this threshold ensures that a sufficient number of individuals are identified as being at risk, thereby maintaining statistical power for subsequent analyses. Equal weights were assigned to all indicators, which is the most common and transparent weighting scheme in the Alkire-Foster approach and avoids bias from subjective weighting (30). DBRI = (1/8) × ΣI_i (where I_i = 0 or 1), with possible values ranging from 0 to 1; higher values indicate higher risk. Following standard Alkire-Foster practice, a cutoff of k = 0.5 was selected to define individuals at high overall risk.

To ensure the robustness of the index, this study calculated, under different cutoff values k (risk thresholds), the number of individuals with dietary-behavioral risk (q = H × n), the total deprivation in dietary-behavioral risk (q × A(k)), the headcount ratio of dietary-behavioral risk H(k), the M(k) of dietary-behavioral risk, and the average deprivation share A(k). The results showed that as k increased, H(k) and M(k) gradually decreased to zero, while A(k) gradually increased to one. At k = 0.5, the rate of change in H(k) was the largest, indicating that the index has good discriminatory power (**Supplementary Table 11**, **Supplementary Figure 2**).

### Statistical analysis

All statistical analyses were performed using R software (version 4.4.2). A two-sided P value < 0.05 was considered statistically significant. Continuous variables were presented as mean (standard deviation), and categorical variables as frequency (%). For group comparisons, normally distributed continuous data were analyzed using the t-test or analysis of variance (ANOVA), non-normally distributed continuous variables using non-parametric tests, and categorical variables using the chi-square (χ²) test. Baseline characteristics were compared between the MetS group and the control group, and the correlations of OLS, DQI-I, and DBRI with MetS were examined. Spearman’s rank correlation coefficient was used to describe the relationships with MetS and with a metabolic health score. Multivariate logistic regression was used to analyze the associations of the four subscores of DQI-I with MetS and its components, with stepwise adjustment for confounders. DBRI was categorized into quartiles, and binary logistic regression was used to examine its association with MetS; a trend test was also performed. The results of the regression analyses are presented as odds ratios (ORs) with 95% confidence intervals (CIs).

## Results

### Comparison of baseline characteristics between the normal group and the MetS group

**Table 1** presents the comparison of general characteristics between the MetS group and the non-MetS group. Regarding sex, the prevalence of MetS was higher in males than in females (28.3% vs. 24.6%, *p* < 0.001). In terms of age, the mean age of the MetS group was higher than that of the normal group (t=-12.663, *p* < 0.001). Significant differences were also observed between the two groups in education level, marital status, occupation, smoking, alcohol consumption, and physical activity (all *p* < 0.05). For physiological indicators, the MetS group had significantly higher values than the non-MetS group in height, weight, WC, HC, SBP, DBP, TC, TG, and LDL-C (all *p* < 0.001), whereas HDL-C was lower in the MetS group than in the normal group (t=25.570, *p* < 0.001).

**Table 1:** Comparison of general characteristics between the MetS group and the normal group (n, %).

Characteristic	Category	MetS group	Normal group	*t/χ* *^2^*	*P*
Gender	Male	1615(28.3)	4085(71.7)	24.105	<0.001
	Female	2112(24.6)	6457(75.4)		
Ethnic group	Han	2379(27.0)	6428(73.0)	9.509	0.002
	Others	1348(24.7)	4114(75.3)		
Educational level	Primary school or below	2625(27.2)	7015(72.8)	19.230	<0.001
	Junior high school	971(23.7)	3127(76.3)		
	Senior high school or above	131(24.7)	400(75.3)		
Marital status	Married	3408(25.7)	9828(74.3)	13.083	<0.001
	Unmarried or divorced	319(30.9)	714(69.1)		
Occupation	Unemployed or retired	691(26.3)	1936(73.7)	72.291	<0.001
	Farmer	2629(27.2)	7047(72.8)		
	Employed	197(16.0)	1031(84.0)		
	Other	210(28.5)	528(71.5)		
Smoking	Never	3142(25.8)	9041(74.2)	4.689	0.030
	Former smoker (quit <10 years)	585(28.0)	1501(72.0)		
Drinking	Never or occasionally	3498(25.9)	10029(74.1)	9.124	0.003
	Everyday	229(30.9)	513(69.1)		
Physical activity	<3 times/week	1180(27.9)	3048(72.1)	9.972	0.002
	≥3 times/week	2547(25.4)	7494(74.6)		
Age, years		58.74 ± 9.48	56.34 ± 10.12	-12.663	<0.001
Height, cm		160.72 ± 8.24	159.66 ± 7.88	-6.976	<0.001
Weight, kg		70.28 ± 10.16	61.77 ± 9.93	-44.714	<0.001
WC, cm		94.09 ± 8.58	84.65 ± 9.03	-55.556	<0.001
HC, cm		97.58 ± 4.82	93.29 ± 5.25	-43.867	<0.001
SBP, mmHg		145.83 ± 18.39	131.88 ± 24.94	-31.269	<0.001
DBP, mmHg		90.00 ± 12.09	81.13 ± 21.44	-23.920	<0.001
TC, mmol/L		5.13 ± 1.75	4.78 ± 0.95	-14.916	<0.001
TG, mmol/L		2.49 ± 1.43	1.43 ± 0.91	-51.638	<0.001
HDL-C, mmol/L		1.22 ± 0.35	1.40 ± 0.39	25.570	<0.001
LDL-C, mmol/L		2.97 ± 1.58	2.79 ± 0.86	-8.593	<0.001

### Associations of OLS, DQI-I, and DBRI with MetS

As shown in **Table 2**, the mean OLS was significantly higher in the MetS group than in the normal group (1.68 ± 0.82 vs. 1.26 ± 0.80, *p* < 0.001). Item-specific analysis showed that the proportions of participants scoring 0 (healthiest) and 1 were higher in the normal group (1,607 [15.2%] and 5,217 [49.5%], respectively) and lower in the MetS group (212 [5.7%] and 1,317 [35.3%], respectively; both *p* < 0.001). The proportions of scores 3 and 4 were similar between the two groups.

**Table 2:** Association of OLS, DQI-I, and DBRI with MetS.

Variable	MetS group (n=3727)	Normal group (n=10542)	P
OLS	1.68 ± 0.82	1.26 ± 0.80	<0.001
0 points	212(5.7%)	1607(15.2%)	<0.001
1 point	1317(35.3%)	5217(49.5%)	
2 points	1757(47.1%)	3137(29.8%)	
3 points	351(9.4%)	486(4.6%)	
4 points	90(2.4%)	95(0.9%)	
DQI-I			
Total score(0-100)	43.32 ± 8.95	43.68 ± 9.08	0.059
Dietary diversity (0-20)	10.71 ± 3.91	10.81 ± 3.91	0.157
Dietary adequacy (0-40)	18.96 ± 5.16	18.96 ± 5.30	0.715
Dietary appropriateness (0-30)	12.47 ± 5.54	12.67 ± 5.65	0.120
Overall balance (0-10)	1.18 ± 1.88	1.24 ± 1.93	0.207
DBRI	0.42 ± 0.15	0.37 ± 0.15	<0.001
DBRI <0.5	2039 (54.7)	7447 (70.6)	<0.001
DBRI ≥0.5	1688 (45.3)	3095 (29.4)	

P for Chi-square test and Mann-Whitney Test.

Regarding DQI-I, neither its total score nor its subscores (dietary diversity, dietary adequacy, dietary moderation, and overall balance) showed significant differences between the two groups (total score *p* = 0.059; dietary diversity *p* = 0.157; dietary adequacy *p* = 0.715; dietary moderation *p* = 0.12; overall balance *p* = 0.207). However, the DBRI showed a statistically significant difference between the two groups (*p* < 0.001): the mean DBRI was 0.42 ± 0.15 in the MetS group and 0.37 ± 0.15 in the normal group. Further analysis revealed that the proportion of participants with DBRI <0.5 was higher in the normal group than in the MetS group (70.6% vs. 54.7%, *p* < 0.001).

### Behavioral risk and MetS risk

#### Correlations of OLS, DQI-I, and DBRI with MetS and metabolic health score

**Figure 2:**
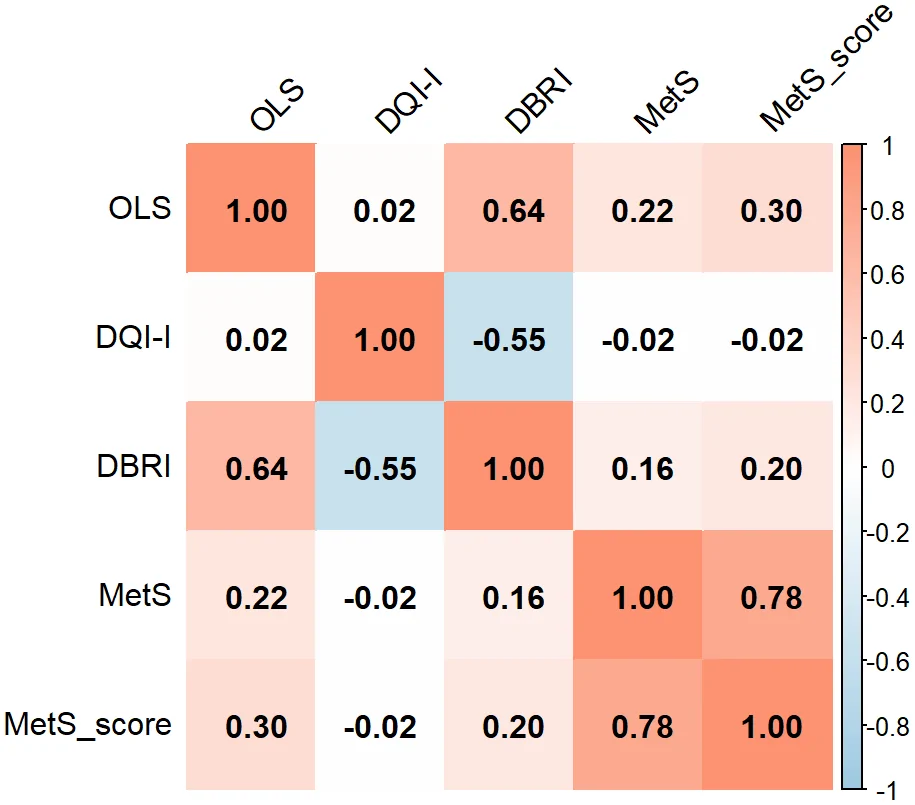
Spearman correlation matrix of OLS, DQI-I, DBRI with MetS and metabolic health score. Numbers are Spearman correlation coefficients. Orange indicates positive correlation, blue indicates negative correlation, with deeper color representing stronger correlation.

To explore the associations of OLS, DQI-I, and DBRI with MetS and the metabolic health score, we first performed correlation analyses. Spearman correlation analysis (**Figure 2**) showed that OLS was positively correlated with MetS and the metabolic health score, with correlation coefficients of 0.22 and 0.30, respectively. In contrast, DQI-I was not significantly correlated with these indicators. For DBRI, the correlation coefficients with MetS and the metabolic health score were 0.16 and 0.20, respectively, which were weaker than those of the obesity-centered lifestyle score alone.

#### Associations of the four DQI-I subscores with MetS and its components

The association results of the four DQI-I subscores with MetS and its components (**Table 3**) were analyzed using multivariate logistic regression models, including Model 1, Model 2, and Model 3.

**Table 3:** Association of the four components of DQI-I with MetS and its components (OR, 95% CI).

Component		MetS	Abnormal WC	Abnormal BP	Abnormal FBG	Abnormal TG	Abnormal HDL-C
Variety	Model 1	0.994 (0.984, 1.003)	1.003 (0.995, 1.012)	0.992 (0.984, 1.000)	1.039 (1.028, 1.050)**	0.980 (0.971, 0.988)**	0.984 (0.972, 0.995)*
	Model 2	0.989 (0.978, 1.000)	1.000 (0.990, 1.010)	1.013 (1.003, 1.023)*	1.048 (1.035, 1.061)**	0.964 (0.954, 0.974)**	0.956 (0.944, 0.970)**
	Model 3	0.991 (0.979, 1.002)	1.000 (0.990, 1.010)	1.016 (1.005, 1.027)*	1.052 (1.038, 1.066)**	0.964 (0.954, 0.975)**	0.964 (0.951, 0.978)**
Adequacy	Model 1	1.000 (0.993, 1.007)	1.007 (1.001, 1.013)*	0.973 (0.967, 0.979)**	1.001 (0.993, 1.009)	1.009 (1.002, 1.015)**	1.021 (1.012, 1.030)**
	Model 2	1.003 (0.994, 1.011)	1.010 (1.002, 1.018)*	0.968 (0.961, 0.976)**	0.977 (0.968, 0.987)**	1.021 (1.013, 1.029)**	1.036 (1.025, 1.046)**
	Model 3	1.001 (1.002, 1.023)*	1.009 (1.001, 1.017)*	0.994 (0.985, 1.002)	0.988 (0.978, 0.998)*	1.018 (1.009, 1.026)*	1.025 (1.014, 1.036)**
Moderation	Model 1	0.994 (0.987, 1.000)	1.006 (1.000, 1.012)	1.004 (0.999, 1.010)	0.979 (0.971, 0.986)**	0.996 (0.990, 1.002)	0.984 (0.976, 0.992)**
	Model 2	0.994 (0.985, 1.002)	1.010 (1.002, 1.017)*	1.000 (0.992, 1.007)	0.981 (0.972, 0.990)**	0.995 (0.987, 1.003)	0.990 (0.980, 1.000)
	Model 3	1.001 (0.992, 1.009)	1.004 (0.996, 1.012)	1.015 (1.007, 1.023)**	0.989 (0.980, 0.999)*	0.993 (0.986, 1.001)	1.004 (0.994, 1.015)
Overall balance	Model 1	0.984 (0.964, 1.003)	1.009 (0.992, 1.026)	0.984 (0.968, 1.001)	0.964 (0.943, 0.986)*	0.998 (0.981, 1.016)	0.970 (0.946, 0.994)*
	Model 2	0.991 (0.967, 1.015)	0.992 (0.972, 1.013)	0.993 (0.973, 1.014)	1.005 (0.978, 1.032)	0.996 (0.925, 1.018)	0.971 (0.942, 1.000)*
	Model 3	0.985 (0.962, 1.010)	0.998 (0.978, 1.020)	0.981 (0.960, 1.003)	1.000 (0.973, 1.027)	0.998 (0.977, 1.020)	0.951 (0.928, 0.987)*

*Model 1, unadjusted; Model 2, mutually adjusted for the four components; Model 3, additionally adjusted for age, sex, ethnicity, education level, marital status, and occupation. MetS, metabolic syndrome; WC, waist circumference; BP, blood pressure; FBG, fasting blood glucose; TG, triglycerides; HDL-C, high-density lipoprotein cholesterol. *P < 0.05; *P < 0.001.

For Variety, Model 1 showed a borderline significant association with MetS (OR = 0.994, 95%CI: 0.984-1.003). In Models 2 and 3, higher Variety scores were associated with increased risks of abnormal BP and FBG, but with significantly reduced risks of abnormal TG and HDL-C.Regarding Adequacy, Model 1 showed no significant association with MetS. In Models 2 or 3, higher Adequacy scores were significantly associated with increased risks of MetS, abnormal WC, abnormal TG, and abnormal HDL-C, but with a decreased risk of abnormal FBG (OR = 0.977, 95%CI: 0.968-0.987).For Moderation, no significant association with MetS was observed. In Model 3, higher Moderation scores were associated with an increased risk of abnormal BP (OR = 1.015, 95%CI: 1.007-1.023), but with a significantly decreased risk of abnormal FBG (OR = 0.964, 95%CI: 0.943-0.986).For Overall balance, no significant association with MetS was observed. In Model 3, higher Overall balance scores were associated with a decreased risk of abnormal HDL-C (OR = 0.951, 95%CI: 0.928-0.987).

#### Association between DBRI and risk of MetS

**Figure 3:**
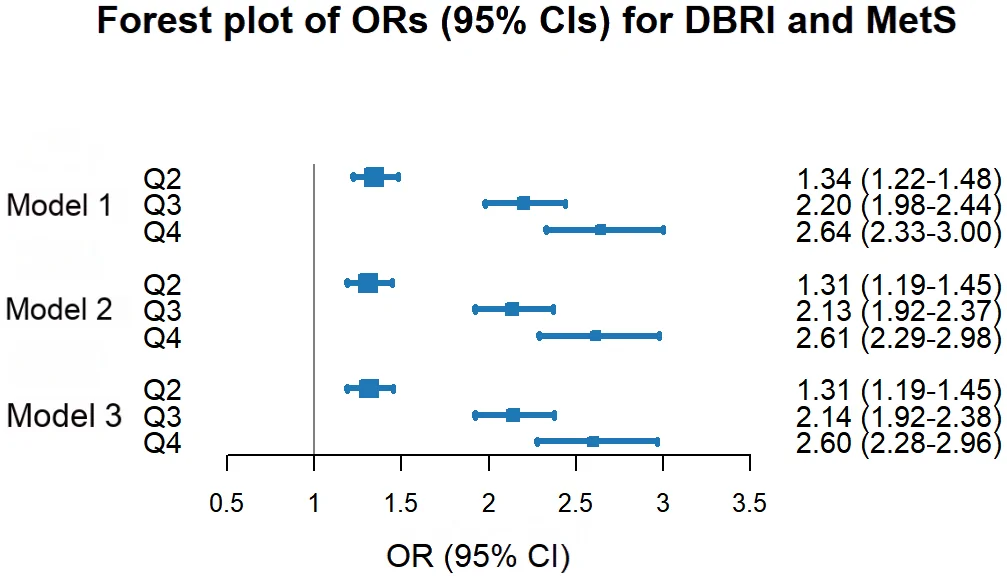
Association between the dietary-behavioral risk index and the risk of metabolic syndrome (MetS). Model 1: unadjusted. Model 2: adjusted for age, sex, ethnicity, education level, marital status, and occupation. Model 3: further adjusted for history of chronic diseases including coronary heart disease and stroke based on Model 2.

Participants were divided into quartiles (Q1, Q2, Q3, Q4) based on DBRI scores, with Q1 as the reference, to examine the association with MetS. The forest plot (**Figure 3**) shows the association between DBRI quartiles and MetS risk. In Model 1 (unadjusted), DBRI was significantly and positively associated with MetS in a dose-response manner (Q4 vs. Q1: OR = 2.64, 95%CI: 2.33-3.00). After adjusting for demographic variables (Model 2), the association remained significant (Q4 vs. Q1: OR = 2.61, 95%CI: 2.29-2.98). After further adjustment for history of coronary heart disease and stroke (Model 3), the association remained robust (Q4 vs. Q1: OR = 2.60, 95%CI: 2.28-2.96). The p for trend across the three models was <0.001, indicating a stable positive association between DBRI and MetS. The association did not change materially after stepwise adjustment for demographic characteristics and unrelated chronic disease history, suggesting that DBRI has good discriminatory ability for MetS.

### Dose-response relationship between DBRI and metabolic syndrome

**Figure 4:**
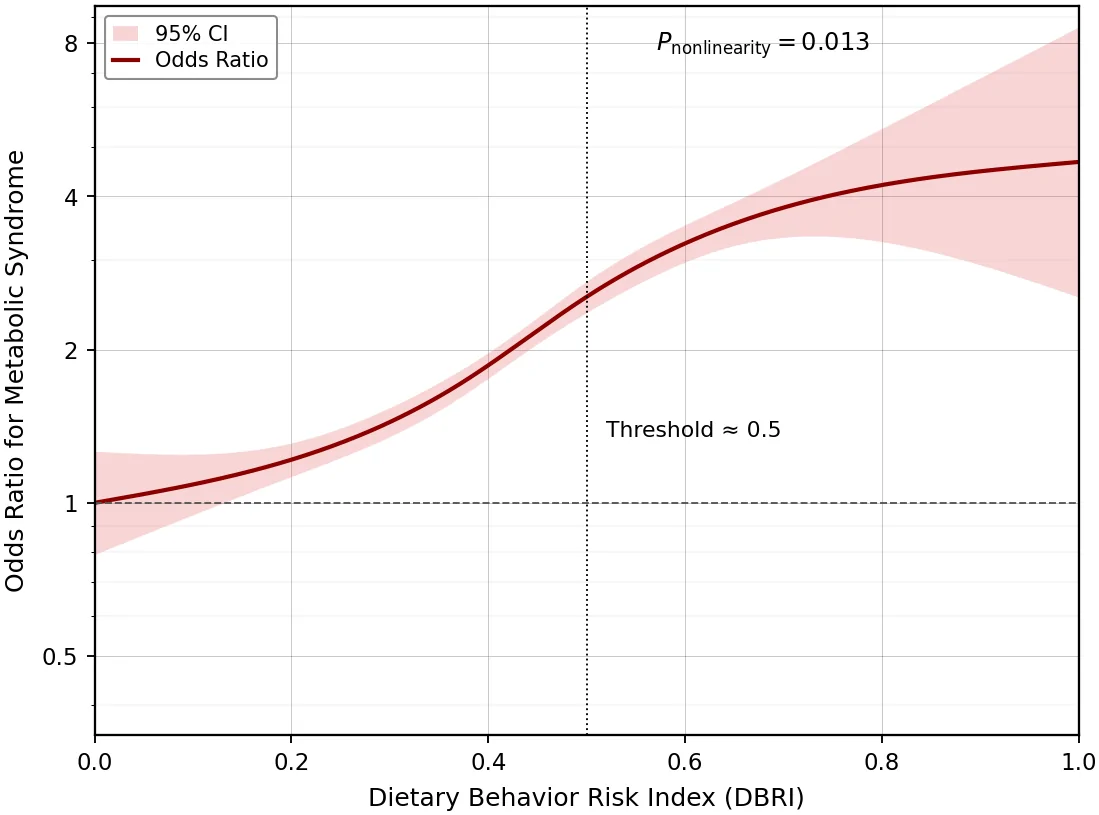
Restricted cubic spline analysis of the association between DBRI and metabolic syndrome (MetS). The solid line represents the odds ratio (OR), and the shaded area indicates the 95% confidence interval. The model was adjusted for age and sex. P for nonlinearity = 0.013.

Restricted cubic spline (RCS) regression, adjusted for age and sex, was used to examine the dose-response relationship between DBRI and the risk of metabolic syndrome (MetS) (**Figure 4**). The results showed a significant nonlinear association between DBRI and MetS risk (P for nonlinearity = 0.013). Using DBRI = 0 as the reference (OR = 1.00), the OR at DBRI = 0.5 was 2.53 (95% CI: 2.36-2.71), and the OR at DBRI = 0.6 was 3.23 (95% CI: 2.97-3.51). These findings suggest that a DBRI value ≥0.5 may serve as a threshold for identifying individuals at high risk for metabolic syndrome.

### Subgroup analysis of the association between DBRI and metabolic syndrome

Subgroup analysis (**Table 3**) showed that the association between DBRI and metabolic syndrome was statistically significant across all subgroups (*p* < 0.001), with OR values ranging from 1.24 to 1.29 in the sex subgroups, 1.25 to 1.32 in the age subgroups, and 1.25 to 1.32 in the education level subgroups. Interaction tests revealed no significant interaction effects for sex (*p* = 0.167), age (*p* = 0.341), or education level (*p* = 0.321), indicating that the association between DBRI and metabolic syndrome was consistent across subgroups.

## Discussion

In this population from the Northwest China Natural Population Cohort, we examined the associations of obesity-centered lifestyle score (OLS), the Diet Quality Index-International (DQI-I), and the Dietary-Behavioral Risk Index (DBRI) with metabolic syndrome (MetS). The results showed that OLS was positively associated with MetS risk, indicating that unhealthier lifestyles were linked to a higher risk of MetS. Although the total DQI-I score was not significantly associated with MetS, its different subscores showed heterogeneous effects on blood pressure, blood glucose, and other MetS components. As a composite index integrating lifestyle and dietary quality, DBRI was significantly and positively associated with MetS in a dose-response manner, and this association remained robust after adjusting for demographic factors and unrelated chronic disease histories. Further analysis revealed a non-linear relationship between DBRI and MetS, with a threshold effect near DBRI≈0.5; beyond this cut-off, the risk increased sharply. The effect of DBRI was stable across various subgroups. However, its association with MetS was not stronger than that of the single lifestyle score, and no clear synergistic interaction between lifestyle and dietary quality was observed.

In this study, we observed a clear positive association of OLS with MetS and the metabolic health score, highlighting the central role of lifestyle interventions in the prevention and control of MetS in rural populations (8). Among the lifestyle factors, body weight was the predominant contributor to MetS (**Appendix Table 8**)(**Supplementary Table 4**), which is consistent with the findings of Yang et al. in a multi-ethnic elderly Chinese population, where higher BMI was significantly associated with MetS prevalence and abnormalities in its individual components, suggesting that weight control is a key strategy for preventing MetS (31). In addition, other lifestyle factors such as physical activity, alcohol consumption, and smoking also had important effects on MetS. Early studies by Haskell and Saris et al. demonstrated that even moderate increases in physical activity were associated with reduced metabolic risk (32, 33), and subsequent research has confirmed these findings (34–36). A recent review further indicated that physical activity improves all clinical components of MetS; for example, aerobic training can reduce systolic blood pressure by 8–12 mmHg and diastolic blood pressure by 5–6 mmHg, and exercise confers metabolic benefits even in the absence of significant weight loss (37). Mechanistically, regular exercise alleviates insulin resistance by promoting glucose uptake in skeletal muscle and improving insulin signaling pathway activity (38). In our study, the proportions of alcohol consumption and smoking were relatively low, and no significant associations of these factors with MetS were observed. This does not contradict previous findings, as the effects of alcohol and smoking on MetS are often not independent but rather interact with other lifestyle factors (39–43). Moreover, attention should be paid to the complex relationships between other unhealthy lifestyle behaviors and metabolic health indicators. For instance, sedentary behavior is associated with insulin resistance and MetS, whereas regular physical activity is linked to improved lipid profiles and a lower risk of hypertension (42–45).

In this study, the overall association between the total DQI-I score and MetS or the metabolic health score was not significant, which is not entirely consistent with previous studies reporting a significant inverse association between diet quality and MetS (46). This discrepancy may be related to the generally low diet quality and small inter-individual variation in the study population(**Supplementary Table 7**). Nevertheless, some associations were observed between specific DQI-I components and individual MetS components, suggesting the complexity of dietary quality in relation to metabolic health and the possible confounding effect of other factors (47). It is noteworthy that the mean total DQI-I score in this population was below 50 out of 100, indicating widespread inadequacies in dietary diversity, adequacy, moderation, and overall balance among rural residents in this area. The generally low diet quality and small inter-individual variation may be statistical reasons for the non-significant overall association, but this finding itself reveals a more serious reality—a public health problem of poor diet quality is prevalent in this population. Low diet quality is not only a risk factor for metabolic syndrome but may also exacerbate the burden of hypertension, diabetes, and cardiovascular diseases through long-term cumulative effects. Therefore, improving overall diet quality in this population should be considered an urgent public health intervention target. Although the total DQI-I score was not significantly associated with MetS, enhancing diet quality may still be of great importance for reducing the risk of MetS. The association between higher dietary adequacy and increased MetS risk may be partly explained by reverse causality—individuals with existing metabolic abnormalities may be more likely to increase their consumption of healthy foods, as commonly reported in cross-sectional dietary surveys (48). In addition, the limited overall variability in dietary quality in this population may also have constrained the ability of the DQI-I to detect true associations. Therefore, this finding should be interpreted with caution, and its causal nature warrants further investigation in prospective studies.

For example, a prospective cohort study in Iran found that a higher Global Diet Quality Score (GDQS) was significantly associated with a 20% lower risk of incident MetS (HR = 0.80, 95% CI: 0.73-0.89), and higher intake of healthy food components was associated with lower incidence of MetS components such as waist circumference and blood glucose (46). Further analysis in our study showed that poor dietary adequacy was significantly associated with an increased risk of MetS. Similarly, Cantero et al. (49) reported that among individuals with MetS, the lowest dietary diversity group had a 28.56-fold higher risk of inadequate nutrient intake compared with the highest diversity group (OR = 28.56). These findings reinforce the importance of healthy lifestyles and good dietary habits in preventing MetS and provide direct evidence that improving lifestyle habits and diet quality can reduce the risk of MetS. From a biological mechanism perspective, unhealthy dietary patterns characterized by high saturated fat and low dietary fiber can induce chronic low-grade inflammation and lead to gut microbiota dysbiosis with reduced production of short-chain fatty acids. These two processes promote each other, jointly exacerbating insulin resistance and overall metabolic abnormalities (50, 51). Of note, because diet as an exposure factor is highly complex, accurately assessing the association between diet and MetS may require more sophisticated statistical methods (e.g., machine learning modeling, latent variable models) or longer-term follow-up studies to reveal subtle relationships (52, 53). Future studies should evaluate this association over larger sample sizes and longer time spans, and may use the Chinese Dietary Balance Index (DBI) to assess dietary intake from three dimensions (insufficient intake, excessive intake, and overall imbalance) (54), thereby further elucidating the potential mechanisms by which dietary factors affect the heterogeneous components of metabolic syndrome, while also focusing on the long-term effects of specific dietary pattern components on the risk of MetS (55).

Studies have demonstrated that combined lifestyle factors (including but not limited to smoking, alcohol consumption, physical activity, and diet quality) influence the risk of cardiovascular disease, cancer, and all-cause mortality (18, 53–55). However, traditional healthy lifestyle scores tend to focus on behavioral dimensions, have limited integration of diet quality, and rarely consider the generally low diet quality and small inter-individual variation characteristic of rural populations (56). The DBRI is essentially constructed from two dimensions - lifestyle risk and diet quality risk - using the Alkire-Foster dual cut-off approach. By capturing the cumulative effects of multidimensional risks, this method can effectively discriminate risk levels in rural populations where overall diet quality is low and individual variation is small, with DBRI≈0.5 serving as a clear risk threshold, thereby comprehensively evaluating the combined effect of multiple risk factors. The present study showed a significant positive dose-response association between DBRI and MetS. Similarly, Li et al. (57) using the Dongfeng-Tongji cohort found that combined lifestyle factors were closely associated with metabolic health in middle-aged and older adults; Lee (58) also confirmed a significant impact of combined lifestyle factors on MetS in Korean men. Furthermore, our study observed differences in DBRI by sex and education level, with men and those with lower education having higher scores. This is consistent with the findings of Ye et al. (23) in southern China, where the association between lifestyle and MetS was more pronounced in men, suggesting that MetS intervention strategies should be tailored by sex and educational background. Based on these findings, DBRI may theoretically assist in risk assessment for MetS in rural settings: preliminary screening can be performed by calculating DBRI using a short questionnaire, risk stratification can be guided by the cut-point of 0.5, and the main sources of individual risk can be identified by examining the separate scores for lifestyle and diet quality dimensions. In summary, the DBRI integrates lifestyle and dietary information and provides a more comprehensive risk profile than the lifestyle score alone, with its distribution varying across different population subgroups (**Supplementary Figure 4**). Its applicability in new populations and predictive value remain to be further validated.

Furthermore, this study found that the combination of abnormal waist circumference and abnormal blood pressure was the most common form of two-component clustering in metabolic syndrome, with abnormal waist circumference playing a central role(**Supplementary Figure 6**). This phenotypic finding is highly consistent with genetic evidence: van Walree et al. (59) demonstrated that waist circumference-related genetic loci show the highest overlap with the shared genetic factors of metabolic syndrome, providing genetic evidence that abdominal obesity is a core driver of metabolic syndrome. Therefore, early intervention targeting abdominal obesity in this population may represent an important breakthrough in halting the progression of metabolic syndrome (60).

This cross-sectional study, based on a large-scale natural population cohort of 14,269 individuals, provides insights into the combined effects of lifestyle and diet quality on MetS and offers a reference for the tertiary prevention of MetS and other major chronic diseases. Furthermore, by incorporating a multidimensional obesity-centered lifestyle score and a diet quality index, we innovatively constructed the Dietary-Behavioral Risk Index (DBRI) using the Alkire-Foster dual cut-off approach. The DBRI captures the combined effects of lifestyle and dietary quality, overcoming the limitation of previous studies that relied on single factors and could not reflect an individual’s comprehensive lifestyle. This provides a new perspective for understanding the roles of lifestyle and dietary factors in MetS risk. In addition, we employed a stratified cluster sampling method and implemented rigorous quality control in data collection, laboratory testing, and statistical analysis, thereby enhancing the reliability of the findings.

Several limitations of this study should be acknowledged. First, the cross-sectional design precludes the inference of causal relationships between lifestyle factors and metabolic syndrome; nevertheless, our findings provide important clues for future prospective studies. Second, lifestyle and dietary information were collected via self-reported questionnaires, which may introduce recall bias. In addition, despite adjusting for major confounders, the inherent nature of observational studies makes it difficult to completely exclude residual confounding. Third, the study participants were recruited from Northwest China (CNC-NX cohort), whose dietary patterns may differ from those in other regions. Thus, caution is needed when extrapolating our findings. However, as this region represents a typical rural area in western China, our findings still offer useful reference for this region and populations with similar dietary patterns. Fourth, the DBRI was constructed using equal weights. Although this approach follows the standard Alkire-Foster framework, alternative weighting strategies might yield different results. Future studies could explore data-driven or theory-driven weighting approaches to further examine the robustness of the DBRI. Finally, although the DBRI offers a new perspective for MetS risk assessment, its external validity requires further verification. Future studies should adopt prospective cohort designs to validate the temporal association between DBRI and MetS, and conduct randomized controlled trials to evaluate intervention effects stratified by a DBRI threshold of ≥0.5. Moreover, the biological mechanisms through which lifestyle and diet quality influence MetS—such as dietary inflammation and adipokine regulation—as well as the modifying effects of individual differences on these associations, warrant in-depth exploration.

## Conclusion

This study revealed a clear positive association of OLS with MetS risk and metabolic health score. Although the total DQI-I score was not significantly associated with MetS, its subscores showed heterogeneous associations with different metabolic components. As a composite indicator integrating lifestyle and diet quality, DBRI was significantly and positively associated with MetS, exhibiting a nonlinear dose-response relationship (threshold at DBRI≈0.5). DBRI demonstrated a certain degree of discriminative ability for identifying MetS (AUC = 0.599), though its performance was not superior to that of the single lifestyle score. No clear synergistic interaction between lifestyle and diet quality was observed for MetS. Single-dimensional dietary assessment may obscure the differential effects of individual components, further highlighting the complexity of lifestyle and dietary factors on metabolic health. Therefore, public health interventions should focus on precise optimization of dietary structure and comprehensive promotion of obesity-related lifestyle habits, and should develop individualized long-term strategies tailored to distinct metabolic abnormalities, in order to more effectively reduce the burden of metabolic syndrome.

## Data Availability

The raw data supporting the conclusions of this article will be made available by the authors, without undue reservation.
