# Supplementary material for: Association of the dietary-behavioral risk index with metabolic syndrome and its components: a cross-sectional study in rural China
Source: Front Endocrinol (Lausanne). 2026 Sep 4;17:1895259. doi: 10.3389/fendo.2026.1895259 (PMC13585529; doi:10.3389/fendo.2026.1895259)
Supplement: Supplementary file 1 [file SupplementaryFile1.docx]

***Supplementary Material***

**Supplementary methods**

***Definition of variables***

Smoking was defined as smoking more than 1 cigarette per day or accumulated over a lifetime, alcohol used was defined as having consumed a variety of alcohol in the past year or at least once a week, and physical activity was defined as exercising more than 30 minutes at least three times a week or 150 minutes a week in your spare time.

***The method of physical examination***

Blood pressure were taken from the subjects in the morning while they were fasting and wearing light clothing. Height and weight were determined using a weight scale (Omron, China). Body mass index (BMI) was calculated using weight and height to assess obesity. Blood pressure was measured three times in the right arm while participants were resting and seated, using an electronic sphygmomanometer (Omron HEM 7301-IT, China).

***List of* contents*:***

**Figure S1** Schematic diagram of the sample size calculation process in this study.

**Table S1** Baseline characteristics of study participants (CNC-NX, 2018-2019).

**Table S2** Baseline characteristics of included and excluded participants.

**Table S3** Lifestyle characteristics and corresponding scores of study participants.

**Table S4** Association of individual lifestyle factors with metabolic syndrome (OR, 95%CI).

**Table S5** Association between lifestyle score and metabolic syndrome (MetS).

**Table S6** Composition and percentage of the Diet Quality Index-International (DQI-I) score.

**Table S7** Diet Quality Index-International (DQI-I) Score and Its Components.

**Table S8** Association of DQI-I quartiles with metabolic syndrome and its components (OR, 95% CI).

**Table S9** Construction framework of the Dietary-Behavioral Risk Index (DBRI).

**Table S10** Collinearity diagnostics of the indicator system.

**Table S11** Results of the Dietary-Behavioral Risk Index (DBRI) measurement among local residents (n=14,269).

**Figure S2** Changes of H(k), M(k), and A(k) for dietary behavioral risk with varying k values.

**Table S12** Contribution rates of indicators to the Dietary-Behavioral Risk Index (DBRI) stratified by MetS status (k = 0.5).

**Figure S3** Distribution of the Dietary-Behavioral Risk Index (DBRI) among study participants.

**Figure S4** Distribution differences of DBRI across population characteristics.

**Figure S5** Distribution differences of DBRI between metabolic syndrome and its components.

**Table S13** Associations of baseline lifestyle score, diet quality index, and DBRI with metabolic health score.

**Table S14** Association between DBRI and metabolic syndrome (MetS).

**Figure S6** Clustering of abnormal components of metabolic syndrome (MetS) in the study population.

**Figure S7** ROC curves of OLS, DQI-I, and DBRI for predicting metabolic syndrome.

**Table S15** Multiplicative interaction analysis of OLS and DQI-I on metabolic syndrome (multivariate logistic regression).

**Table S16** Additive interaction analysis of OLS and DQI-I on metabolic syndrome.

**Table S17** Logistic regression analysis of DBRI quartiles with metabolic syndrome (IDF criteria): a sensitivity analysis.

**Figure S1 Schematic diagram of the sample size calculation process in this study.** The horizontal axis (N) represents the sample size, and the vertical axis represents the odds ratio (OR). P0 indicates the assumed prevalence of metabolic syndrome (MetS) at 12%. The statistical power is set at 0.9, with α = 0.05.

**Table S1 Baseline characteristics of study participants** **(CNC-NX, 2018-2019)**

| Variable | Total | Male | Female | *t/χ^2^* | *P* |
| --- | --- | --- | --- | --- | --- |
| N | 14269 | 5700 | 8569 |  |  |
| Age (years) | 56.97±10.01 | 58.87±9.85 | 55.70±9.91 | 18.804 | <0.001 |
| Ethnicity, n (%) |  |  |  |  |  |
| Han | 8807(61.7) | 3597(63.1) | 5210(60.8) | 7.696 | 0.006 |
| Others | 5462(38.3) | 2103(36.9) | 3359(39.2) |  |  |
| Education level, n (%) |  |  |  |  |  |
| Primary school or below | 9640(67.6) | 3499(61.4) | 6141(71.7) | 184.221 | <0.001 |
| Junior high school | 4098(28.7) | 1902(33.4) | 2196(25.6) |  |  |
| Senior high school or above | 531(3.7) | 299(5.2) | 232(2.7) |  |  |
| Marital status, n (%) |  |  |  |  |  |
| Married | 13236(92.8) | 5409(94.9) | 7827(91.3) | 64.379 | <0.001 |
| Unmarried / divorced / widowed | 1033(7.2) | 291(5.1) | 742(8.7) |  |  |
| Occupation, n (%) |  |  |  |  |  |
| Unemployed / retired | 2627(18.4) | 675(11.8) | 1952(22.8) | 352.003 | <0.001 |
| Farmer | 9676(67.8) | 4002(70.2) | 5674(66.2) |  |  |
| Employed (non-agricultural) | 1228(8.6) | 625(11.0) | 603(7.0) |  |  |
| Others | 738(5.2) | 398(7.0) | 340(4.0) |  |  |
| History of hypertension, n (%) |  |  |  |  |  |
| No | 7181(50.3) | 2714(47.6) | 4467(52.1) | 27.922 | <0.001 |
| Yes | 7088(49.7) | 2986(52.4) | 4102(47.9) |  |  |
| History of diabetes, n (%) |  |  |  |  |  |
| No | 12866(90.2) | 5124(89.9) | 7742(90.4) | 0.709 | 0.400 |
| Yes | 1398(9.8) | 573(10.1) | 825(9.6) |  |  |
| History of coronary heart disease, n (%) |  |  |  |  |  |
| No | 13371(93.7) | 5422(95.1) | 7949(92.8) | 32.279 | <0.001 |
| Yes | 898(6.3) | 278(4.9) | 620(7.2) |  |  |

**Table S2** **Baseline characteristics of included and excluded participants**

| Characteristic | Included group (n=14,269) | Excluded group (n=1,533) | *t/χ^2^* | *P* |
| --- | --- | --- | --- | --- |
| Sex, n (%) |  |  |  |  |
| Male | 5700(39.9) | 634(41.4) | 1.146 | 0.284 |
| Female | 8569(60.1) | 899(58.6) |  |  |
| Ethnicity, n (%) |  |  |  |  |
| Han | 8807(61.7) | 1012(66.0) | 10.845 | <0.001 |
| Other ethnicities | 5462(38.3) | 521(34.0) |  |  |
| Education level, n (%) |  |  |  |  |
| Primary school or below | 9640(67.6) | 1007(65.7) | 4.972 | 0.083 |
| Junior high school | 4098(28.7) | 453(29.5) |  |  |
| Senior high school or above | 531(3.7) | 73(4.8) |  |  |
| Marital status, n (%) |  |  |  |  |
| Married | 13236(92.8) | 1409(91.9) | 1.471 | 0.225 |
| Unmarried/divorced/ widowed | 1033(7.2) | 124(8.1) |  |  |
| Occupation, n (%) |  |  |  |  |
| Unemployed / retired | 2627(18.4) | 194(12.7) | 41.421 | <0.001 |
| Farmer | 9676(67.8) | 1149(75.0) |  |  |
| Employed (non-agricultural) | 1228(8.6) | 105(6.8) |  |  |
| Others | 738(5.2) | 85(5.5) |  |  |
| Smoking status, n (%) |  |  |  |  |
| Never | 12183(85.4) | 1285(83.8) | 2.670 | 0.102 |
| Current (quit <10 years) | 2086(14.6) | 248(16.2) |  |  |
| Alcohol consumption, n (%) |  |  |  |  |
| Never or occasional | 13527(94.8) | 1450(94.6) | 0.128 | 0.720 |
| Daily | 742(5.2) | 83(5.4) |  |  |
| Physical activity, n (%) |  |  |  |  |
| Active (≥3 times/week) | 4228(29.6) | 402(26.2) | 7.759 | <0.001 |
| Inactive (<3 times/week) | 10041(70.4) | 1131(73.8) |  |  |
| Diabetes history, n (%) |  |  |  |  |
| No | 12866(90.2) | 850(87.4) | 7.666 | 0.005 |
| Yes | 1398(9.8) | 122(12.6) |  |  |
| Coronary heart disease history, n (%) |  |  |  |  |
| No | 13371(93.7) | 1425(93.1) | 0.923 | 0.337 |
| Yes | 898(6.3) | 106(6.9) |  |  |
| Stroke history, n (%) |  |  |  |  |
| No | 14017(98.2) | 1492(97.5) | 4.668 | 0.031 |
| Yes | 252(1.8) | 39(2.5) |  |  |
| Age (years), mean ± SD | 56.97±10.01 | 56.98±10.62 | -0.028 | 0.978 |
| Height (cm), mean ± SD | 159.93±7.99 | 161.28±8.52 | -5.180 | <0.001 |
| Weight (kg), mean ± SD | 63.99±10.67 | 64.25±10.79 | -0.753 | 0.452 |
| WC (cm), mean ± SD | 87.11±9.83 | 86.29±9.83 | 2.588 | 0.010 |
| HC(cm), mean ± SD | 94.41±5.47 | 94.61±10.13 | -1.032 | 0.302 |
| SBP (mmHg), mean ± SD | 135.53±24.20 | 137.34±21.27 | -2.556 | 0.011 |
| DBP (mmHg), mean ± SD | 83.45±19.82 | 83.73±12.87 | -0.491 | 0.624 |
| FPG(mmol/L), mean ± SD | 5.70±2.58 | 6.11±3.15 | -0.634 | 0.448 |
| TC (mmol/L), mean ± SD | 4.87±1.22 | 4.78±1.71 | 1.332 | 0.096 |
| TG (mmol/L), mean ± SD | 1.71±1.17 | 1.55±0.96 | 4.258 | <0.001 |
| HDL (mmol/L), mean ± SD | 1.35±0.39 | 1.37±0.43 | -1.336 | 0.182 |
| LDL (mmol/L), mean ± SD | 2.84±1.09 | 2.83±0.90 | 0.118 | 0.906 |

**Table S3 Lifestyle characteristics and corresponding scores of study participants**

| Variable | Total (n=14269) | Male (n=5700) | Female (n=8569) | *t/χ^2^* | *P* value |
| --- | --- | --- | --- | --- | --- |
| Smoking |  |  |  |  |  |
| No | 12183(85.4) | 3722(65.3) | 8461(98.7) | 3066.894 | <0.001 |
| Yes | 2086(14.6) | 1978(34.7) | 108(1.3) |  |  |
| Drinking |  |  |  |  |  |
| No | 13527(94.8) | 5020(88.1) | 8507(99.3) | 872.000 | <0.001 |
| Yes | 742(5.2) | 680(11.9) | 62(0.7) |  |  |
| Overweight or obese |  |  |  |  |  |
| No | 7565(53.0) | 2969(52.1) | 4596(53.6) | 3.291 | 0.070 |
| Yes | 6704(47.0) | 2731(47.9) | 3973(46.4) |  |  |
| Inactive physical exercise |  |  |  |  |  |
| No | 4228(29.6) | 1800(31.6) | 2428(28.3) | 17.279 | <0.001 |
| Yes | 10041(70.4) | 3900(68.4) | 6141(71.7) |  |  |
| OLS | 1.37±0.827 | 1.63±0.947 | 1.20±0.684 | 31.425 | <0.001 |
| 0 points | 1819(12.7) | 566(9.9) | 1253(14.6) | 1466.942 | <0.001 |
| 1 point | 6534(45.8) | 2122(37.2) | 4412(51.5) |  |  |
| 2 points | 4894(34.3) | 2051(36) | 2843(33.2) |  |  |
| 3 points | 837(5.9) | 779(13.7) | 58(0.7) |  |  |
| 4 ponits | 185(1.3) | 182(3.2) | 3(0) |  |  |

Abbreviations: OLS, Obesity‑Centered Lifestyle Score.

**Table S4 Association of individual lifestyle factors with metabolic syndrome (OR, 95%CI)**

| Parameter | Smoking | Drinking | Overweight/Obesity | Physical inactivity |
| --- | --- | --- | --- | --- |
| Model 1 |  |  |  |  |
| OR (95% CI) | 1.121 (1.010, 1.243) | 1.280 (1.088, 1.501) | 6.008 (5.514, 6.553) | 0.878 (0.810, 0.952) |
| *P* | 0.030 | 0.003 | <0.001 | 0.002 |
| Model 2 |  |  |  |  |
| OR (95% CI) | 1.147 (1.019, 1.290) | 1.062 (0.885, 1.273) | 5.998 (5.504, 6.543) | 0.916 (0.840, 1.000) |
| *P* | 0.023 | 0.516 | <0.001 | 0.048 |
| Model 3 |  |  |  |  |
| OR (95% CI) | 1.232 (1.058, 1.435) | 1.019 (0.820, 1.264) | 6.322 (5.719, 6.997) | 0.949 (0.856, 1.053) |
| *P* | 0.007 | 0.863 | <0.001 | 0.323 |

Note: Model 1 was unadjusted. Model 2 was mutually adjusted for the four lifestyle factors (smoking, drinking, overweight/obesity, and physical inactivity). Model 3 was additionally adjusted for age, sex, ethnicity, education level, marital status, occupation, and history of diabetes, hypertension, and coronary heart disease based on Model 2.

**Table S5 Association between lifestyle score and metabolic syndrome (MetS)**

| Parameter | Parameter | | |
| --- | --- | --- | --- |
|  | 0～1 points | 2 points | 3～4 points |
| n (%) | 8353 (58.5) | 4894 (34.3) | 1022 (7.2) |
| Model 1 (unadjusted) |  |  |  |
| OR (95% CI) | Reference | 2.500 (2.306, 2.710) | 3.388 (2.957, 3.879) |
| *P* |  | <0.001 | <0.001 |
| *P* for trend | <0.001 | | |
| Model 2 |  |  |  |
| OR (95% CI) | Reference | 2.683 (2.440, 2.952) | 4.412 (3.705, 5.255) |
| *P* |  | <0.001 | <0.001 |
| *P* for trend | <0.001 | | |

Note: Model 1 was unadjusted. Model 2 was adjusted for age, sex, ethnicity, education level, marital status, occupation, history of diabetes, history of hypertension, and history of coronary heart disease.

**Table S6 Composition and percentage of the Diet Quality Index-International (DQI-I) score**

| Components | Full marks | Scoring criteria | | Percentage (%) |
| --- | --- | --- | --- | --- |
|  |  | Score | Standard |  |
| Variety | 0-20 |  |  |  |
| Diversity of food composition (livestock, poultry, fish and eggs; milk and beans; cereals; fruits; vegetables) | 0-15 | 15 | The intake quantity of each type of food exceeds 1 serving per day. | 6.4 |
|  |  | 12 | Any type of food is less than 1 serving per day | 14.3 |
|  |  | 9 | Any two types of food are less than 1 serving per day | 49.9 |
|  |  | 6 | Any three types of food with less than 1 serving per day | 22.8 |
|  |  | 3 | Any of the four food groups is less than 1 serving per day | 6.5 |
|  |  | 0 | Less than 1 serving per day for each type of food | 0.0 |
| Diversity of protein sources (livestock meat; poultry; aquatic products; eggs; milk and dairy products; beans and bean products) | 0-5 | 5 | Protein ≥3 different food sources | 16.9 |
|  |  | 3 | Protein sources from 2 different foods | 20.9 |
|  |  | 1 | Protein sources from 1 different foods | 57.3 |
|  |  | 0 | None | 4.9 |
| Adequacy | 0-40 |  |  |  |
| Vegetable group* | 0-5 | 5 | ≥100% recommended intake | 6.2 |
|  |  | 3 | 50%-100% recommended intake | 19.2 |
|  |  | 1 | <50% recommended intake | 74.5 |
|  |  | 0 | 0% recommended intake | 0.0 |
| Fruit group* | 0-5 | 5 | ≥100% recommended intake | 1.1 |
|  |  | 3 | 50%-100% recommended intake | 8.4 |
|  |  | 1 | <50% recommended intake | 56.5 |
|  |  | 0 | 0% recommended intake | 34.0 |
| Cereals and grains* | 0-5 | 5 | ≥100% recommended intake | 3.3 |
|  |  | 3 | 50%-100% recommended intake | 81.8 |
|  |  | 1 | <50% recommended intake | 14.8 |
|  |  | 0 | 0% recommended intake | 0.0 |
| Dietary fiber* | 0-5 | 5 | ≥100% recommended intake | 3.3 |
|  |  | 3 | 50%-100% recommended intake | 28.6 |
|  |  | 1 | <50% recommended intake | 68.1 |
|  |  | 0 | 0% recommended intake | 0.0 |
| Protein | 0-5 | 5 | ≥100% recommended intake | 70.3 |
|  |  | 3 | 50%-100% recommended intake | 29.5 |
|  |  | 1 | <50% recommended intake | 0.2 |
|  |  | 0 | 0% recommended intake | 0.0 |
| Iron^#^ | 0-5 | 5 | ≥100% recommended intake | 65.4 |
|  |  | 3 | 50%-100% recommended intake | 29.4 |
|  |  | 1 | <50% recommended intake | 5.2 |
|  |  | 0 | 0% recommended intake | 0.0 |
| Calcium^#^ | 0-5 | 5 | ≥100% recommended intake | 1.1 |
|  |  | 3 | 50%-100% recommended intake | 10.9 |
|  |  | 1 | <50% recommended intake | 88.0 |
|  |  | 0 | 0% recommended intake | 0.0 |
| Vitamin C^#^ | 0-5 | 5 | ≥100% recommended intake | 13.1 |
|  |  | 3 | 50%-100% recommended intake | 29.3 |
|  |  | 1 | <50% recommended intake | 57.6 |
|  |  | 0 | 0% recommended intake | 0.0 |
| Moderation | 0-30 |  |  |  |
| Total Fat | 0-6 | 6 | ≤20% Proportion of energy supply | 5.6 |
|  |  | 3 | 20%-30% Proportion of energy supply | 33.7 |
|  |  | 0 | >30% Proportion of energy supply | 60.7 |
| Saturated fat | 0-6 | 6 | ≤7% Proportion of energy supply | 85.4 |
|  |  | 3 | 7%-10% Proportion of energy supply | 12.4 |
|  |  | 0 | >10% Proportion of energy supply | 2.2 |
| Cholesterol | 0-6 | 6 | ≤300 mg/d | 52.9 |
|  |  |  | 300-400 mg/d | 9.5 |
|  |  |  | >400 mg/d | 37.5 |
| Sodium | 0-6 | 6 | ≤2400 mg/d | 13.6 |
|  |  |  | 2400-3400 mg/d | 19.8 |
|  |  |  | >3400 mg/d | 66.5 |
| Pure energy food | 0-6 | 6 | ≤3% Proportion of energy supply | 2.2 |
|  |  | 3 | 3%-10% Proportion of energy supply | 25.3 |
|  |  | 0 | >10% Proportion of energy supply | 72.5 |
| Overall balance | 0-10 |  |  |  |
| Energy supply ratio of the three major macronutrients (carbohydrates: proteins: fats) | 0-6 | 6 | 55-65: 10-15: 15-25 | 7.8 |
|  |  | 4 | 52-68: 9-16: 13-27 | 7.1 |
|  |  | 2 | 50-70: 8-17: 12-30 | 11.3 |
|  |  | 0 | Other | 73.8 |
| Fatty acid energy supply ratio(PUFA+MUFA/SFA) | 0-4 | 4 | P/S=1-1.5 and M/S=1-1.5 | 1.6 |
|  |  | 2 | P/S=0.8-1.7 and M/S=0.8-1.7 | 9.3 |
|  |  | 0 | Other | 89.1 |

Note: * indicates values based on different energy levels: 7118 kJ (1700 kcal)/9211 kJ (2200 kcal)/11304 kJ (2700 kcal); the recommended standard for protein is that the proportion of energy it provides exceeds 10%; # indicates that the scoring system is based on China’s RNI values.
Abbreviations: DQI‑I, Diet Quality Index‑International.

**Table S7 Diet Quality Index-International (DQI-I) Score and Its Components**

| Components | Score | Mean | SD |
| --- | --- | --- | --- |
| DQI-I, total score | 0-100 | 43.59 | 9.05 |
| Variety | 0-20 | 10.78 | 3.91 |
| Diversity of food composition | 0-15 | 8.74 | 2.82 |
| Diversity of protein sources | 0-5 | 2.05 | 1.58 |
| Adequacy | 0-40 | 18.96 | 5.26 |
| Vegetable group | 0-5 | 1.63 | 1.17 |
| Fruit group | 0-5 | 0.87 | 0.91 |
| Cereals and grains | 0-5 | 2.77 | 0.82 |
| Dietary fiber | 0-5 | 1.70 | 1.08 |
| Protein | 0-5 | 4.40 | 0.92 |
| Iron | 0-5 | 4.21 | 1.17 |
| Calcium | 0-5 | 1.26 | 0.74 |
| Vitamin C | 0-5 | 2.11 | 1.43 |
| Moderation | 0-30 | 12.61 | 5.62 |
| Total Fat | 0-6 | 1.35 | 1.80 |
| Saturated fat | 0-6 | 5.50 | 1.28 |
| Cholesterol | 0-6 | 3.46 | 2.82 |
| Sodium | 0-6 | 1.41 | 2.17 |
| Pure energy food | 0-6 | 0.89 | 1.51 |
| Overall balance | 0-10 | 1.22 | 1.92 |
| The energy supply ratio of the three major macronutrients | 0-6 | 0.97 | 1.85 |
| Fatty acid energy supply ratio | 0-4 | 0.25 | 0.75 |

**Table S8 Association of DQI-I quartiles with metabolic syndrome and its components (OR, 95% CI)**

| Variable |  | Q1 | Q2 | Q3 | Q4 | P for trend |
| --- | --- | --- | --- | --- | --- | --- |
| MetS | Model 1 | Reference | 0.991 (0.892, 1.101) | 0.916 (0.827, 1.015) | 0.928 (0.836, 1.029) | 0.071 |
|  | Model 2 |  | 1.035 (0.931, 1.151) | 0.985 (0.887, 1.093) | 1.037 (0.932, 1.093) | 0.711 |
| WC abnormality | Model 1 | Reference | 1.017 (0.927, 1.116) | 1.029 (0.940, 1.126) | 1.128 (1.029, 1.235)* | 0.014 |
|  | Model 2 |  | 1.001 (0.911, 1.099) | 1.006 (0.918, 1.102) | 1.091 (0.944, 1.198) | 0.098 |
| BP abnormality | Model 1 | Reference | 0.902 (0.822, 0.990)* | 0.786 (0.718, 0.860)** | 0.821 (0.749, 0.899)** | <0.001 |
|  | Model 2 |  | 1.026 (0.930, 1.132) | 0.978 (0.888, 1.076) | 1.123 (1.018, 1.238)* | 0.043 |
| FBG abnormality | Model 1 | Reference | 1.070 (0.952, 1.202) | 1.004 (0.896, 1.126) | 1.008 (0.898, 1.131) | 0.877 |
|  | Model 2 |  | 1.136 (1.009, 1.278)* | 1.110 (0.988, 1.247)* | 1.172 (1.041, 1.319)* | 0.010 |
| TG abnormality | Model 1 | Reference | 1.026 (0.932, 1.129) | 0.956 (0.870, 1.049) | 0.961 (0.875, 1.057) | 0.233 |
|  | Model 2 |  | 1.007 (0.914, 1.109) | 0.927 (0.843, 1.019) | 0.928 (0.843, 1.022) | 0.038 |
| HDL-C abnormality | Model 1 | Reference | 0.994 (0.874, 1.130) | 0.981 (0.866, 1.111) | 0.908 (0.799, 1.032) | 0.156 |
|  | Model 2 |  | 0.991 (0.869, 1.130) | 0.946 (0.832, 1.075) | 0.899 (0.787, 1.027) | 0.101 |

Note: Model 2 was adjusted for age, sex, ethnicity, education level, marital status, and occupation. Data are presented as odds ratio (OR) with 95% confidence interval (CI). **P < 0.05; **P < 0.001 (compared with the reference group Q1).

Abbreviations: DQI-I, Diet Quality Index-International; MetS, metabolic syndrome; WC, waist circumference; BP, blood pressure; FBG, fasting blood glucose; TG, triglycerides; HDL-C, high-density lipoprotein cholesterol; Q1–Q4, quartiles of DQI-I score (Q1 as reference).

**Table S9 Construction framework of the Dietary-Behavioral Risk Index (DBRI)**

| Dimension | Indicator | Deprivation cutoff | Weight^a^ |
| --- | --- | --- | --- |
| Lifestyle risk |  |  |  |
|  | Alcohol consumption | Current smoking = 1; otherwise = 0 | 1/8 |
|  | Smoking | Heavy drinking = 1; otherwise = 0 | 1/8 |
|  | Overweight or obesity | BMI>25 = 1；otherwise= 0 | 1/8 |
|  | Physical inactivity | Physical exercise < 3 times/week = 1; otherwise = 0 | 1/8 |
| Diet quality risk^b^ |  |  |  |
|  | Variety | Score<10 = 1；otherwise = 0 | 1/8 |
|  | Adequacy | Score<17 = 1；otherwise = 0 | 1/8 |
|  | Moderation | Score<12 = 1；otherwise = 0 | 1/8 |
|  | Overall balance | Score<2 = 1；otherwise = 0 | 1/8 |

Note: a Equal weights were assigned to each dimension. b The deprivation cutoff for diet quality was defined as the 40th percentile (P40) of the corresponding score.

Abbreviations: BMI, body mass index; DBRI, Dietary-Behavioral Risk Index.
Abbreviations: OLS, Obesity‑Centered Lifestyle Score; DQI‑I, Diet Quality Index‑International; DBRI, Dietary‑Behavioral Risk Index.

**Table S10 Collinearity diagnostics of the indicator system**

| Indicator | Collinearity Diagnostics | |
| --- | --- | --- |
|  | Tolerance | VIF |
| Dimension 1 |  |  |
| Alcohol consumption | 0.902 | 1.108 |
| Smoking | 0.897 | 1.115 |
| Overweight or obesity | 0.996 | 1.004 |
| Insufficient physical activity | 0.985 | 1.015 |
| Dimension 2 |  |  |
| Variety | 0.979 | 1.022 |
| Adequacy | 0.827 | 1.210 |
| Moderation | 0.789 | 1.267 |
| Overall balance | 0.891 | 1.122 |

**Table S11 Results of the Dietary-Behavioral Risk Index (DBRI) measurement among local residents (n=14,269)**

| K value | Number of dietary-behavioral risks q=H·n | Total deprivation q×A_(K)_ | Incidence of dietary-behavioral risks H_(k)_ | Dietary-behavioral risk intensity M_(k)_ | Average deprivation share A_(k)_ |
| --- | --- | --- | --- | --- | --- |
| 0.125 | 14069 | 5416.565 | 0.986 | 0.380 | 0.385 |
| 0.250 | 12899 | 5275.691 | 0.904 | 0.369 | 0.409 |
| 0.375 | 9702 | 4472.622 | 0.680 | 0.313 | 0.461 |
| 0.500 | 4780 | 2624.220 | 0.355 | 0.184 | 0.549 |
| 0.625 | 1512 | 988.848 | 0.106 | 0.070 | 0.654 |
| 0.750 | 328 | 249.280 | 0.023 | 0.017 | 0.760 |
| 0.875 | 28 | 24.640 | 0.002 | 0.001 | 0.880 |
| 1.000 | － | － | － | － | 1.000 |

**Figure S2 Changes of H(k), M(k), and A(k) for dietary behavioral risk with varying k values.** The horizontal axis represents the k value. H(k) indicates the incidence of dietary behavioral risk; M(k) indicates the dietary behavioral risk; A(k) indicates the average deprivation share.

**Table S12 Contribution rates of indicators to the Dietary-Behavioral Risk Index (DBRI) stratified by MetS status (k = 0.5)**

| Dimension | Indicator | Total population | | MetS group | | Non-MetS group | |
| --- | --- | --- | --- | --- | --- | --- | --- |
|  |  | Indicator contribution rate% | Dimension contribution rate% | Indicator contribution rate% | Dimension contribution rate% | Indicator contribution rate% | Dimension contribution rate% |
| Lifestyle risk | |  |  |  |  |  |  |
|  | Alcohol consumption | 6.6 | 45.8 | 6.1 | 48.4 | 6.9 | 44.3 |
|  | Smoking | 2.8 |  | 2.6 |  | 2.9 |  |
|  | Overweight or obesity | 16.7 |  | 20.8 |  | 14.4 |  |
|  | Insufficient physical activity | 19.7 |  | 18.9 |  | 20.1 |  |
| Dietary quality risk | |  |  |  |  |  |  |
|  | Variety | 10.1 | 54.2 | 9.1 | 51.6 | 10.7 | 55.7 |
|  | Adequacy | 13.1 |  | 13.1 |  | 13.1 |  |
|  | Moderation | 10.9 |  | 10.0 |  | 11.4 |  |
|  | Overall balance | 20.1 |  | 19.4 |  | 20.4 |  |
| H | | 0.335 | | 0.453 | | 0.294 | |
| M | | 0.184 | | 0.251 | | 0.160 | |

### Note: Data are presented as contribution rates (%). k = 0.5 indicates the dual cut-off value used in the Alkire-Foster approach. The contribution rate of each indicator is the proportion of that indicator's weighted deprivation share to the total DBRI. The dimension contribution rate is the sum of indicator contribution rates within that dimension

**Figure S3 Distribution of the Dietary-Behavioral Risk Index (DBRI) among study participants.**

**Figure S4 Distribution differences of DBRI across population characteristics.**

**Figure S5 Distribution differences of DBRI between metabolic syndrome and its components.**

**Table S13 Associations of baseline lifestyle score, diet quality index, and DBRI with metabolic health score**

| Variable | Metabolic health score | | | | | | *P* | Test for Linear Trend |
| --- | --- | --- | --- | --- | --- | --- | --- | --- |
|  | 0 points | 1 points | 2 points | 3 points | 4 points | 5 points |  |  |
| Lifestyle score |  |  |  |  |  |  |  |  |
| 0 points | 105(32.8) | 145(33.0) | 116(27.6) | 54(22.2) | 15(23.4) | 1(20.0) | <0.001 | <0.001 |
| 1 points | 160(50.0) | 172(39.1) | 175(41.6) | 104(42.8) | 27(42.2) | 1(20.0) |  |  |
| 2 points | 41(12.8) | 96(21.8) | 98(23.3) | 66(27.2) | 15(23.4) | 0 |  |  |
| 3 points | 14(4.4) | 23(5.2) | 25(5.9) | 17(7.0) | 5(7.8) | 2(40.0) |  |  |
| 4 points | 0 | 4(0.9) | 7(1.7) | 2(0.8) | 2(3.1) | 1(20.0) |  |  |
| Diet Quality Index-International (DQI-I) |  |  |  |  |  |  |  |  |
| Total DQI-I score | 49.70±6.28 | 49.45±5.90 | 49.22±6.47 | 49.39±5.89 | 49.95±6.17 | 48.60±6.77 | 0.883 | 0.139 |
| Dietary variety | 13.88±3.41 | 13.95±3.45 | 13.57±3.47 | 13.89±3.51 | 13.89±3.60 | 10.80±2.59 | 0.117 | 0.515 |
| Dietary adequacy | 20.72±3.42 | 20.32±3.34 | 20.51±3.40 | 20.44±3.25 | 21.06±3.49 | 22.8±3.90 | 0.303 | 0.371 |
| Dietary moderation | 14.15±2.85 | 14.10±2.70 | 14.15±2.95 | 14.06±2.85 | 14.06±2.67 | 13.8±3.42 | 0.998 | 0.537 |
| Overall balance | 0.98±1.62 | 1.07±1.67 | 0.98±1.60 | 1.01±1.67 | 0.94±1.42 | 1.20±1.79 | 0.945 | 0.04 |
| DBRI | 0.32±0.15 | 0.33±0.17 | 0.35±0.17 | 0.35±0.16 | 0.35±0.19 | 0.53±0.29 | 0.008 | <0.001 |
| DBRI <0.5 | 258(80.6) | 336(76.4) | 304(72.2) | 172(70.8) | 46(71.9) | 2(40.0) | 0.020 | 0.001 |
| DBRI ≥0.5 | 62(19.4) | 104(23.6) | 117(27.8) | 71(29.2) | 18(28.1) | 3(60.0) |  |  |

P for Chi-square test and Mann-Whitney Test

**Table S14 Association between DBRI and metabolic syndrome (MetS)**

| **Parameter** | **DBRI** | | | |
| --- | --- | --- | --- | --- |
|  | **Q1** | **Q2** | **Q3** | **Q4** |
| n (%) | 4562 (32.0) | 4924 (34.5) | 3266 (22.9) | 1517 (10.6) |
| Model 1 |  |  |  |  |
| OR (95% CI) | Reference | 1.341(1.215,1.481) | 2.201(1.984,2.441) | 2.642(2.326,3.001) |
| *P* |  | ＜0.001 | ＜0.001 | ＜0.001 |
| *P* for trend | <0.001 | | | |
| Model 2 |  |  |  |  |
| OR (95% CI) |  | 1.31(1.186,1.448) | 2.134(1.921,2.371) | 2.61(2.289,2.975) |
| *P* |  | ＜0.001 | ＜0.001 | ＜0.001 |
| P for trend | <0.001 | | | |
| Model 3 |  |  |  |  |
| OR (95% CI) | Reference | 1.313(1.188，1.451) | 2.138(1.924，2.375) | 2.598(2.279，2.963) |
| P |  | ＜0.001 | ＜0.001 | ＜0.001 |
| P for trend | <0.001 | | | |

Note: Model 1 was adjusted for no covariates. Model 2 was adjusted for age, sex, ethnicity, education level, marital status, and occupation. Model 3 was further adjusted for history of coronary heart disease, stroke, and other chronic diseases based on Model 2.

**Figure S6 Clustering of abnormal components of metabolic syndrome (MetS) in the study population.**

**Figure S7 ROC curves of OLS, DQI-I, and DBRI for predicting metabolic syndrome.** Receiver operating characteristic (ROC) curves comparing the predictive performance of obesity-centered lifestyle score (OLS), diet quality index-international (DQI-I), and dietary behavior risk index (DBRI) for metabolic syndrome (MetS) among rural Chinese adults. The area under the curve (AUC) values were 0.636 for OLS, 0.490 for DQI-I, and 0.599 for DBRI. Pairwise comparisons of AUCs were performed using the DeLong method: OLS vs. DBRI (p = 0.023), OLS vs. DQI-I (p < 0.001), and DBRI vs. DQI-I (p = 0.001). The diagonal dashed line represents the reference line (AUC = 0.50). **Table S15** Multiplicative interaction analysis of OLS and DQI-I on metabolic syndrome (multivariate logistic regression)

| ariable | B | SE | P | OR (95% CI) |
| --- | --- | --- | --- | --- |
| Age | 0.031 | 0.002 | <0.001 | 1.031 (1.027-1.035) |
| Sex (male vs. female) | 0.205 | 0.042 | <0.001 | 1.228 (1.130-1.334) |
| OLS | 0.513 | 0.152 | 0.001 | 1.671 (1.241-2.250) |
| DQI‑I_rev | -0.005 | 0.005 | 0.331 | 0.995 (0.986-1.005) |
| OLS× DQI‑I_rev | 0.003 | 0.003 | 0.28 | 1.003 (0.998-1.008) |
| Constant | -3.858 | 0.305 | <0.001 | 0.021 |

Note: The model was adjusted for age and sex. The product term of OLS and DQI‑I showed a P value of 0.280 with an OR of 1.003 (95% CI: 0.998-1.008), indicating no significant multiplicative interaction between the two factors.

**Table S16** Additive interaction analysis of OLS and DQI-I on metabolic syndrome

| Variable | β | SE | P | OR (95% CI) |
| --- | --- | --- | --- | --- |
| Age | 0.028 | 0.002 | <0.001 | 1.028 (1.024-1.032) |
| Sex (male vs. female) | 0.075 | 0.041 | 0.067 | 1.078 (0.995-1.168) |
| Combined group (reference: low OLS + low DQI-I) |  |  |  |  |
| Low OLS + high DQI-I | -0.049 | 0.057 | 0.389 | 0.952 (0.851-1.065) |
| High OLS + low DQI-I | 0.97 | 0.055 | <0.001 | 2.637 (2.369-2.936) |
| High OLS + high DQI-I | 1.01 | 0.056 | <0.001 | 2.746 (2.460-3.065) |
| Constant | -3.209 | 0.15 | <0.001 | 0.04 |

Note: Participants were classified into four groups based on the medians of OLS and DQI‑I. The reference group was “low OLS + low DQI‑I”. The model was adjusted for age and sex. Additive interaction measures: relative excess risk due to interaction (RERI) = 0.157, attributable proportion (AP) = 0.057, synergy index (S) = 1.099, indicating no significant additive interaction.

**Table S17** Logistic regression analysis of DBRI quartiles with metabolic syndrome (IDF criteria): a sensitivity analysis

| Parameter | DBRI | | |  | |
| --- | --- | --- | --- | --- | --- |
|  | Q1 | Q2 | Q3 | Q4 | P for trend |
| n (%) | 4562 (32.0) | 4924 (34.5) | 3266 (22.9) | 1517 (10.6) |  |
| Model 1 |  |  |  |  | ＜0.001 |
| OR (95% CI) | Reference | 1.316(1.213，1.429) | 1.937(1.769，2.122) | 2.129(1.892，2.396) |  |
| P |  | ＜0.001 | ＜0.001 | ＜0.001 |  |
| Model 2 |  |  |  |  | ＜0.001 |
| OR (95% CI) | Reference | 1.3(1.196，1.412) | 1.965(1.791，2.156) | 2.401(2.126，2.713) |  |
| P |  | ＜0.001 | ＜0.001 | ＜0.001 |  |
| Model 3 |  |  |  |  | ＜0.001 |
| OR (95% CI) | Reference | 1.301 (1.198，1.414) | 1.966 (1.792，2.158) | 2.394 (2.119，2.705) |  |
| P |  | ＜0.001 | ＜0.001 | ＜0.001 |  |

Note: DBRI was categorized into quartiles (Q1, Q2, Q3, Q4), with Q1 as the reference group. Data are presented as odds ratios (OR) with 95% confidence intervals (CI). Model 1 was unadjusted. Model 2 adjusted for age, sex, ethnicity, education level, marital status, and occupation. Model 3 further adjusted for history of chronic diseases including coronary heart disease and stroke. The results indicate that the association between DBRI and MetS remained significant across all models, with a clear dose–response trend..
